# Protective Effects of Sodium Selenite against Aflatoxin B_1_-Induced Oxidative Stress and Apoptosis in Broiler Spleen

**DOI:** 10.3390/ijerph10072834

**Published:** 2013-07-09

**Authors:** Fengyuan Wang, Gang Shu, Xi Peng, Jing Fang, Kejie Chen, Hengmin Cui, Zhengli Chen, Zhicai Zuo, Junliang Deng, Yi Geng, Weimin Lai

**Affiliations:** Key Laboratory of Animal Diseases and Environmental Hazards of Sichuan Province, College of Veterinary Medicine, Sichuan Agricultural University, Ya’an 625014, China; E-Mails: wfy_sccd@163.com (F.W.); sug119@163.com (G.S.); ckj930@126.com (K.C.); cui580420@sicau.edu.cn (H.C.); chzhli75@163.com (Z.C.); zzcjl@126.com (Z.Z.); dengjl213@126.com (J.D.); gengyisicau@126.com (Y.G.); nwm_mm2004@163.com (W.L.)

**Keywords:** aflatoxin b_1_, sodium selenite, oxidative stress, apoptosis, spleen

## Abstract

The aim of this study was to investigate the possible protective role of sodium selenite on aflatoxin B_1_-induced oxidative stress and apoptosis in spleen of broilers. Two hundred one-day-old male broilers, divided into five groups, were fed with basal diet (control group), 0.3 mg/kg AFB_1_ (AFB_1_ group), 0.3 mg/kg AFB_1_ + 0.2 mg/kg Se (+Se group I), 0.3 mg/kg AFB_1_ + 0.4 mg/kg Se (+Se group II) and 0.3 mg/kg AFB_1_ + 0.6 mg/kg Se (+Se group III), respectively. According to biochemical assays, AFB_1_ significantly decreased the activities of glutathione peroxidase, total superoxide dismutase, glutathione reductase, catalase and the level of glutathione hormone, while it increased the level of malondialdehyde. Moreover, AFB_1_ increased the percentage of apoptosis cells by flow cytometry and the occurrence of apoptotic cells by TUNEL assay. Simultaneous supplementation with sodium selenite restored these parameters to be close to those in control group. In conclusion, sodium selenite exhibited protective effects on AFB_1_-induced splenic toxicity in broilers by inhibiting oxidative stress and excessive apoptosis.

## 1. Introduction

Aflatoxin B_1_ (AFB_1_) is a fungal toxin produced by a species of *Aspergillus*, mainly by *Aspergillus flavus*, and is a common dietary contaminant all over the World, mostly in the hot and humid climate regions [1]. The toxic and carcinogenic effects of AFB_1_ are intimately linked with its biotransformation [2]. The active intermediate, AFB_1_-exo-8,9-epoxide, can bind with DNA to form the predominant *trans*-8, 9-dihydro-8-(*N*^7^-guanyl)-9-hydroxy-AFB_1_ (AFB_1_-*N*^7^-Gua) adduct which causes DNA lesions [3]. Acute or chronic aflatoxicosis in chicken results in decreased meat/egg production and growth rates, negative feed conversions, immunosuppression, and increased susceptibility to other diseases [4,5,6]. AFB_1_ is able to induce reactive oxygen species (ROS) generation which causes oxidative stress, leading to oxidation of proteins, lipids and DNA [7]. Meanwhile, AFB_1_ is able to be a direct and indirect initiator as well as promoter of genotoxicity and apoptotic process [8].

Selenium (Se) was recognized only 40 years ago as being an essential element in the nutrition of animals and humans, and an essential component of a number of enzymes [9]. Se has recognized antioxidant properties [10] and functions as a redox centre, for instance, the family of selenium-dependent glutathione peroxidases could reduce hydrogen peroxide, lipid and phospholipid hydroperoxides to harmless products [11]. Miller (2001) reported that small increases in concentration of sodium selenite can confer highly significant protection against oxidative damage [12].

The spleen is the principal peripheral lymphoid organ and plays an important role in protective immune reactions [13]. It is involved in humoral and cellular immune responses through its role in the generation, maturation and storage of lymphocytes [14]. Previous study revealed that AFB_1_ significantly affected the development of spleen in ducklings [15], and Se could ameliorate the negative effects induced by AFB_1_ [16]. In order to investigate the effects of sodium selenite against AFB_1_-induced oxidative stress and apoptosis in spleen, splenic glutathione peroxidase (GSH-Px), total superoxide dismutase (SOD), glutathione reductase (GR) and catalase (CAT) activities as well as glutathione hormone (GSH) and malondialdehyde (MDA) contents were detected by biochemical methods, and the apoptosis of splenocytes was determined by flow cytometry and a TUNEL assay.

## 2. Materials and Methods

### 2.1. Animals and Diets

Two hundred one-day-old male avian broilers (weighing 45 ± 5 g) were purchased from Wenjiang poultry farm (Sichuan Province, China) and randomly divided into five equal groups of 40 each and fed on diets as follows: control group, AFB_1_ group (0.3 mg/kg AFB_1_), +Se group I (0.3 mg/kg AFB_1_ + 0.2 mg/kg Se), +Se group II (0.3 mg/kg AFB_1_ + 0.4 mg/kg Se) and +Se group III (0.3 mg/kg AFB_1_ + 0.6 mg/kg Se). By hydride-generation atomic absorption spectroscopy, the contents of Se in control group dietary were 0.404 mg/kg. Thus, the concentration of Se in each group was: 0.404 mg/kg (control group), 0.404 mg/kg (AFB_1_ group), 0.604 mg/kg (+Se group I), 0.804 mg/kg (+Se group II) and 1.004 mg/kg (+Se group III), respectively. Aflatoxin B_1_ (AFB_1_) was obtained from Fermentek Ltd (Jerusalem, Israel, 1162-65-8). AFB_1_ farinose solid (3 mg) was completely dissolved in methanol (30 mL), and then the 30 mL mixture was mixed into the 10 kg corn-soybean basal diet to formulate the AFB_1_ diet of experimental groups containing 0.3 mg/kg AFB_1_. The concentration of 0.3 mg/kg AFB_1_ was chosen according to Ghosh’s study [17]. The equivalent methanol was mixed into the corn-soybean basal diet to produced control diet. Then the methanol of diets was evaporated at 98 °F (37 °C). Broilers were provided with drinking water as well as the aforementioned diets *ad libitum* for 21 days. All procedures of the experiment were performed in compliance with laws and guidelines of Sichuan Agriculture University Animal Welfare Institute.

### 2.2. Lipid Peroxidation and Antioxidant Defense System Assays

At 7, 14 and 21 days of the experiment, six chickens in each group were euthanized and the splenic tissues were immediately collected for evaluating state of oxidative stress. Splenic tissue (1 g) was homogenized with normal saline buffer (9 mL) through a cell homogenizer in an ice bath and centrifuged at 3,000 r/min for 10 min to obtain a clear supernatant. The centrifuge used was a TD24-WS of Xiangyi Co. (Changsha, China). After determining the amount of total protein in the supernatant of the splenic homogenate by the method of Bradford [18], the GSH, MDA contents and GSH-Px, SOD, GR, CAT activities in the splenic supernatant were measured by biochemical method following the instruction of reagent kits (Jiancheng, Nanjing, China), as described by Li *et al.* [19]. GSH assays were based on the development of a yellow color when DTNB was added to compounds containing sulfhydryl groups. MDA assays were determined by the thiobarbituric acid (TBA) colorimetric method. GSH-Px activities were detected by the consumption of glutathione. SOD activities were determined by the xanthine oxidase method. GR activities can be monitored by the NADPH consumption. CAT activities were determined by the H_2_O_2_ decomposition rate. The absorbance of the supernatants were measured by spectrophotometric assay at 532 nm for MDA, 412 nm for GSH and GSH-Px, 550 nm for SOD, 340 nm for GR and 240 nm for CAT, the values were expressed as nmol/mg protein for GSH and MDA, and units (U) per mg protein for GSH-Px, SOD, GR and CAT.

### 2.3. Annexin V Apoptosis Detection by Flow Cytometry

At 7, 14 and 21 days of the experiment, six chickens in each group were euthanized and spleens were sampled from each chick to determine the percentage of apoptotic cells by flow cytometry [20]. Briefly, the excised spleens were immediately ground to form a cell suspension and filtered. Then, the cells were washed and suspended in 1 × binding buffer at a concentration of 1 × 10^6^ cells/mL. Annexin V-fluorescein isothiocyanate (V-FITC, 5 µL) and propidium iodide (PI, 5 µL) were added into 100 µL cell suspension, and incubated at 25 °C for 15 min in the dark. 1 × binding buffer (400 µL) was added to the mixture, and then the apoptotic cells were assayed by flow cytometry (BD FACSCalibur) within 1 h. The annexin V-FITC Kit was obtained from BD Pharmingen (Franklin Lakes, NJ, USA, 556547).

### 2.4. TUNEL

The DNA fragmentation indicative of apoptosis was examined using terminal deoxynucleotidyl transferase-mediated dUTP nick end labeling method (TUNEL), that could detect early stage apoptosis and examine the topographic distribution of apoptotic cells [21]. At 7, 14 and 21 days of the experiment, six chickens in each group were euthanized and spleens were sampled and fixed in 4% paraformaldehyde and routinely processed in paraffin. Thin sections (5 ìm) of each tissue were sliced from each block and mounted on glass. Slides were stained with TUNEL assay, which was performed using apoptosis detection kit (Merck, New York, Germany, QIA33) according to the manufacturer’s instructions, as described by Peng *et al.* [22].

### 2.5. Statistical Analysis

The results were shown as means ± standard deviation (M ± SD). Statistical analysis was performed using one-way analysis of variance (ANOVA) of SPSS 16.0 software. The difference between groups was considered significant when a probability (*p*) was <0.05.

## 3. Results

**Figure 1:**
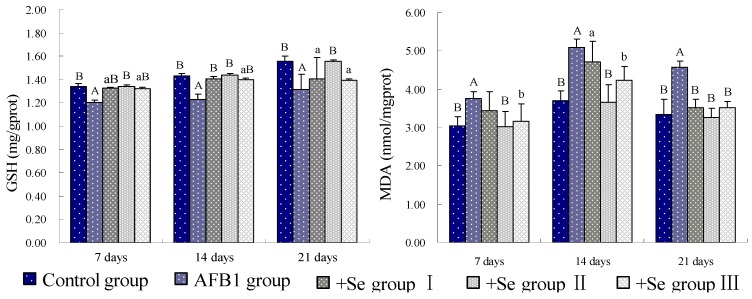
Effect of AFB_1_ and Se on the GSH and MDA contents of spleen in chickens.

**Figure 2:**
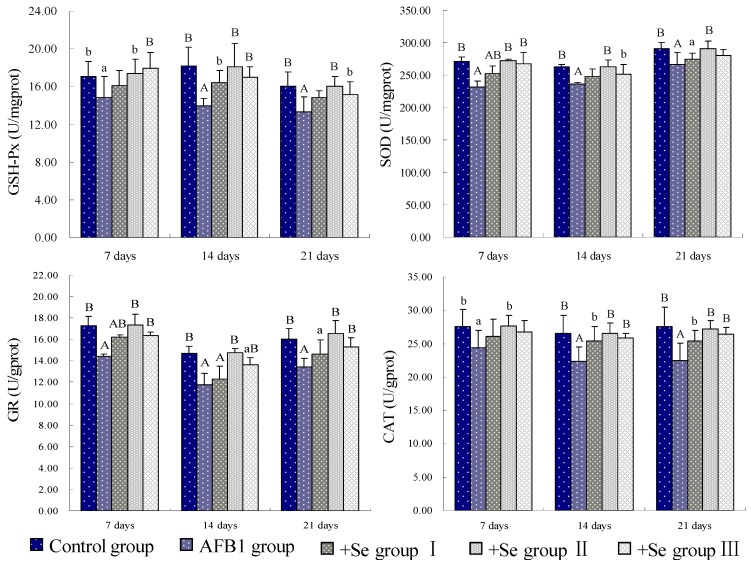
Effect of AFB_1_ and Se on the GSH-Px, SOD, GR, and CAT activities of spleen in chickens.

Compared with the control group, the GSH contents of spleen were significantly decreased (*p* < 0.01) in the AFB_1_ group. However, when compared with those in the AFB_1_ group, the GSH contents of spleen were increased in three +Se groups, especially in the +Se group II, from 7 to 21 days of age. At the same time, the MDA contents were higher in AFB_1_ group than those in control group (*p* < 0.01), while those were lower in three +Se groups than those in AFB_1_ group, especially in +Se group II (Figure 1). As shown in Figure 2, the activities of GSH-Px, SOD, GR and CAT were all decreased (*p* < 0.01 or *p* < 0.05) in the AFB_1_ group when compared with those in control group from 7 to 21 days of age. However, the activities of GSH-Px, SOD, GR and CAT showed an increase in three +Se groups when compared with those in AFB_1_ group, especially in +Se group II.

Data are presented with the means ± standard deviation (n = 5); (a) *p* < 0.05, compared with the control group; (A) *p* < 0.01, compared with the control group; (b) *p* < 0.05, compared with the AFB_1_ group; (B) *p* < 0.01, compared with the AFB_1_ group Data were analyzed by variance analysis using SPSS 16.0 software.

Data are presented with the means ± standard deviation (n = 5); (a) *p* < 0.05, compared with the control group; (A) *p* < 0.01, compared with the control group; (b) *p* < 0.05, compared with the AFB_1_ group; (B) *p* < 0.01, compared with the AFB_1_ group Data were analyzed by variance analysis using SPSS 16.0 software.

**Figure 3:**
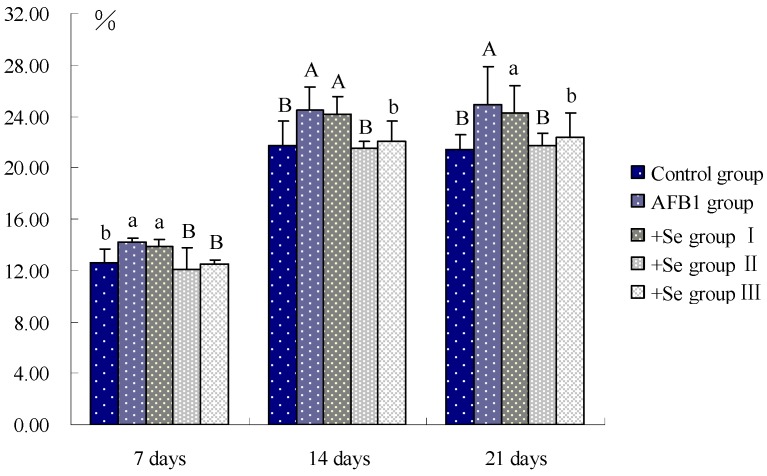
Effect of AFB_1_ and Se on the percentage of apoptotic splenocytes in chickens.

As shown in Figure 3, the percentage of apoptotic splenocytes was increased (*p* < 0.05) in the AFB_1_ group at 7 days of age and significantly increased (*p* < 0.01) at 14 and 21 days of age, when compared with that in control group. However, compared with that in AFB_1_ group, the percentages of apoptotic splenocytes were decreased (*p* < 0.05) in three +Se groups, and significantly decreased (*p* < 0.01) in +Se group II.

**Figure 4:**
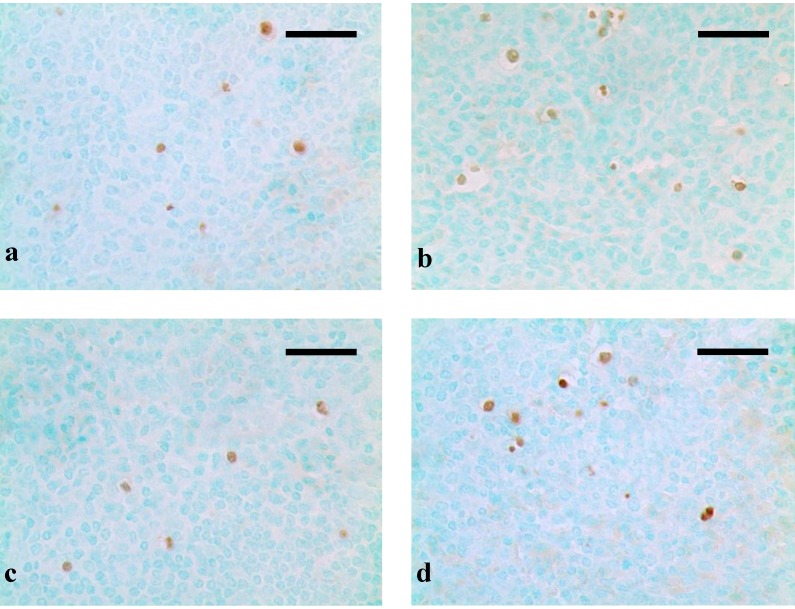
Histological images of TUNEL assay.

Through the TUNEL assay, apoptotic cells with brown-stained nuclei were found in both the red pulp and white pulp of the spleens. The occurrence of apoptotic cells in AFB_1_ groups were increased when compared with those in control group at 21 days (Figure 4a). When Se was added in the dietary, however, the occurrence of apoptotic cells were decreased, especially in the +Se group II (Figure 4b-d).

Data are presented with the means ± standard deviation (n = 5); (a) *p* < 0.05, compared with the control group; (A) *p* < 0.01, compared with the control group; (b) *p* < 0.05, compared with the AFB_1_ group; (B) *p* < 0.01, compared with the AFB_1_ group Data were analyzed by variance analysis using SPSS 16.0 software.

Control group (a); The numbers of apoptotic cells were increased in AFB_1_ group when compared with those in control group (b); The numbers of apoptotic cells were decreased in +Se group II (c) and III (d) when compared with AFB_1_ group, especially in +Se group II (c). bars = 20 μm.

## 4. Discussion and Conclusions

AFB_1_ can cause oxidative damages, which may be one of the underlining mechanisms for AFB_1_-induced cell injury and DNA damage, and eventually lead to tumorigenesis [23]. As previous study revealed, AFB_1_ induced oxidative stress, which included the decrease of the level of GSH and the activities of SOD and GSH-Px, and the increase of level of MDA in lymphocytes of human [24,25], the increase of MDA and lipid hydroperoxide (LHP) in hepatocytes of rats [26]. Our data showed that 0.3 mg/kg dietary AFB_1_ could increase MDA contents, and decrease GSH contents, GSH-Px, SOD, GR and CAT activities, which demonstrated an oxidative stress in spleen of broilers. These results were consistented with previous studies.

As is known, AFB_1_ may cause ROS generation, lipid peroxidation and formation of 8-hydroxydeoxyguanosine (8-OHdG) *in vivo* and *in vitro* [26]. When the concentration of ROS exceeds the antioxidant capability of cells, oxidative stress occurs in a cell or tissue [27]. The levels of enzymatic antioxidants and non-enzymatic antioxidants are the main determinants of the antioxidant defence mechanism of the cell [28]. In our present study, the activities of antioxidase, including GSH-Px, SOD, GR and CAT were all markedly decreased in AFB_1_ groups compared with those of the control group. These enzymatic antioxidants have been recognized to play an important role in the anti-oxidant mechanism of the body, which can eliminate ROS from cell, for instance, SOD converts O_2_·^−^ into H_2_O_2_ and O_2_; CAT and GSH-Px reduces H_2_O_2_ into H_2_O and O_2_ [29]. If their activities decreased, the oxygen free radical would induce harmful effects to biological systems. GSH, a non-enzymatic antioxidant, is also an early biological marker of the oxidative stress [30]. It plays a role in the suppression of oxygen free-radical formation and the reduction in NO generation [31]. As well known, through the action of glutathione-S-transferase, the metabolites of AFB_1_ are mainly conjugated with GSH before to be excreted [32]. So, a decreased content of GSH was observed in AFB_1_ group in our study. The MDA is the end product of lipoperoxydation, considered as a late biomarker of oxidative stress and cellular damage [8]. In the present study, we found an increased level of MDA in the AFB_1_ group, which could result in extensive cell damage and death [33].

Apoptosis is a mode of programmed cell death [34], whereas excessive apoptosis is actively involved in immunosuppression in various circumstances [35]. Several studies indicated that AFB_1_ was able to induce apoptosis in hepatocytes, lung and bone marrow cells, or human bronchial epithelial cells [36,37,38]. In our study, through flow cytometer method and TUNEL assay, an increased apoptotic splenocytes was observed in AFB_1_ groups, which revealed one mechanism of AFB_1_-induced immunosuppression. Previous studies have clarified that oxidative stress would induce mitochondrial dysfunction, nuclear translocation, DNA binding, and transcriptional activity of p53, and then activate the course of cell-cycle arrest and cell apoptosis [39,40]. According to our results, it was concluded that the increased percentage of apoptotic splenocytes was closely related to oxidative stress in the AFB_1_ group.

Se is an essential micronutrient to humans and animals and is required for anti-oxidant selenoenzymes [41], but high concentration of Se is toxic when it excesses the threshold [42]. According to previous study, Se could alleviate the destructive oxidative stress caused by various factors, like heroin, adriamycin, and cisplatin [43,44,45]. Our present study showed that in three +Se groups, the contents of GSH and the activities of GSH-Px, SOD, GR, and CAT were all increased when compared with AFB_1_ group, and the MDA content was decreased. It may be associated with increased antioxidative function resulting from an increase in activity of GSH-Px whose center is Se [46]. As previous study revealed, Se can inhibit lipid peroxidation [47]. Furthermore, Se also has an anti-apoptotic property involved with ROS and mitochondria linked signal pathway [48]. In our study, through flow cytometer method and TUNEL assay, a decreased apoptosis status of splenocytes was observed in three +Se groups. Coincide with previous study [49], our results suggested that adequate GSH levels could reduce ROS formation and protect against apoptosis. Moreover, Se can also prevent from oxidative damage to mitochondria DNA [47], and accordingly inhibit apoptosis induced by mitochondria pathway. However, it was observed in +Se group III that the contents of GSH and the activities of GSH-Px, SOD, GR, CAT were higher than those in +Se group II, and the contents of MDA was lower than those in +Se group II. This may result from the toxicity of excessive Se in diet.

According to our results and the aforementioned discussion, it was concluded that administrated dietary sodium selenite can prevent AFB_1_-induced immunosuppression by inhibiting AFB_1_-induced oxidative stress and excessive apoptosis in spleen of broilers, especially at the concentration of 0.804 mg/kg.
